# Women’s Night in Emergency Medicine Mentorship Program: A SWOT Analysis

**DOI:** 10.5811/westjem.2019.11.44433

**Published:** 2019-12-18

**Authors:** Alison G. Marshall, Priyanka Sista, Katie R. Colton, Abra Fant, Howard S. Kim, Patrick M. Lank, Danielle M. McCarthy

**Affiliations:** * Temple University, Lewis Katz School of Medicine, Department of Emergency Medicine, Philadelphia, Pennsylvania; † Northwestern University, Feinberg School of Medicine, Department of Emergency Medicine, Chicago, Illinois

## Abstract

**Introduction:**

Women in emergency medicine (EM) at all career stages report gender-specific obstacles to satisfaction and advancement. Programs that facilitate longitudinal mentoring, professional development, and networking may ameliorate these barriers.

**Methods:**

We designed and implemented a program for female residents, faculty, and alumnae from our EM training program to enhance social support, leadership training and professional mentorship opportunities. An anonymous, online survey was sent to participants at the end of the academic year, using a SWOT (strengths, weaknesses, opportunities, and threats) format. The survey collected free-text responses designed to evaluate the program.

**Results:**

Of 43 invited participants, 32 responded (74.4%). Eight themes emerged from the free-text responses and were grouped by SWOT domain. We identified four themes relating to the “strength” domain: 1) creating a dedicated space; 2) networking community; 3) building solidarity; and 4) providing forward guidance. Responses to the “weaknesses” and “threats” questions were combined due to overlapping codes and resulted in three themes: 5) barriers to participation; 6) the threat of poorly structured events lapsing into negativity; and 7) concerns about external optics. A final theme: 8) expansion of program scope was noted in the “opportunity” domain.

**Conclusion:**

This program evaluation of the Women’s Night curriculum demonstrates it was a positive addition to the formal curriculum, providing longitudinal professional development opportunities. Sharing the strengths of the program, along with identified weaknesses, threats, and opportunities for advancement allows other departments to learn from this experience and implement similar models that use existing intellectual and social capital.

## BACKGROUND

Gender disparities among emergency physicians (EP) influence compensation, promotion, and wellbeing of female physicians.^1^–^14^ Professional networking, leadership training, and access to mentorship are vital mechanisms for closing the gender gap in academic medicine.^15^–^20^ Yet women are joining the specialty of emergency medicine (EM) without established pathways for professional development to mitigate existing barriers. The disparity of female faculty in academic EM subsequently results in fewer female role models and mentorship opportunities for women, despite the well-established benefit of these relationships in career development and academic productivity.^13^, ^15^–^18^

Increasingly, EM has been host to national movements raising awareness around gender disparities and promoting new platforms in which to address them.^18^–^24^ However, few examples in the literature describe comprehensive departmental residency or post-residency programs that provide opportunities for female physicians to establish mentorship relationships and obtain leadership positions in academic medicine.^17^,^18^,^25^,^26^

## OBJECTIVES

We set out to design and implement a program for female residents, faculty, and local alumnae from our EM training program to enhance social support, leadership training, and professional mentorship opportunities. Once implemented, we sought to evaluate the first year of our “Women’s Night” initiative using a SWOT (strengths, weaknesses, opportunities, threats) analysis.^27^

## CURRICULAR DESIGN

We used a six-step approach to curriculum development, starting with the pre-established problem of gender disparities in academic EM and the subsequent need to proactively address those disparities with enhanced professional development as our problem identification/general needs assessment.^28^ Prospective participants, including female faculty, alumnae and residents were invited to join an ad hoc committee that then completed a targeted, departmental needs assessment through group discussion. A primary goal to “cultivate solidarity and mentorship among female residents, alumnae and faculty through a Women’s Night program” was established. Specific objectives for the Women’s Night program included a) completing a professional development activity at each meeting, and b) maintaining unstructured time for social interaction and networking.

The Women’s Night program launched in 2016 and comprised six evening events. All female residents, faculty and local alumnae were invited. Female residents were scheduled off from clinical duties, with the exception of those on off-service rotations (e.g., intensive care unit), whereas faculty could request the night off. This night was part of any given resident’s time off, and participants worked the same number of shifts as non-participants and male residents.

Faculty or chief residents hosted the 2–3 hour events in their homes or restaurants, and two female faculty members split the cost of each event. Professional development activities were organized by Women’s Night program leaders. (See Appendix for list of topics.) We conducted the SWOT analysis as our evaluation of the program.

## IMPACT & EFFECTIVENESS

### Survey

An end-of-year, institutional review board (IRB)-approved survey to evaluate the program was sent to anyone invited to the events over the prior academic year. Data were collected from June–July 2017 via a REDcap (Research Electronic Data Capture) survey.^29^ Participants completed questions designed to evaluate the program using a SWOT format.^27^,^30^ Our SWOT analysis used a classic four-question template of open-ended questions requesting participant reflection on programmatic strengths, weaknesses, opportunities, and threats. Finally, participants were asked to reflect on whether and how Women’s Night had “influenced” them. Survey questions were drafted by two members of the study team (DMM, AGM) and consensus was reached by iterative review.

### Data Analysis

The study team was comprised of two female attending EPs, two male attending EPs and three female resident EPs. Qualitative content analysis using a consensual qualitative approach was undertaken with a primary coding team (DMM, PS, KC, AGM) generating initial codes and themes. A secondary audit team (PML, AF, HSK) reviewed all original data and thematic structure to evaluate for omissions or oversimplifications.^32^

## RESULTS

Forty-eight participants were invited to Women’s Night events, of whom 35 attended one or more events. Five invitees were ineligible because of study involvement. Of the 43 remaining physicians, 32 (74.4%) completed the survey (24 who had attended events and seven who had not). All respondents were female, with 40.6% residents, 37.5% faculty, and 34.4% alumnae. (Several of the faculty are also program alumnae.)

The primary team arrived at an initial 20 codes, resulting in seven themes. Audit team review identified one additional theme resulting in a final framework of eight themes, presented in the table. Due to overlapping data in the “weaknesses” and “threats” domains, the themes were consolidated. Additionally, responses to the question of how the events “influenced” participants aligned with the “strengths” themes.

### Strengths

Overarching themes inside the “strengths” category were *dedicated space, networking community*, *solidarity, and forward guidance*. Respondents noted that creating a *dedicated space*, provided “protected time” to discuss “tough topics specific to women in EM in a non-judgmental atmosphere,” allowed learning “from other women’s experiences,” and fomented dialogue. This space subsequently facilitated *networking and community* building. Residents particularly mentioned *forward guidance* noting “women’s night has inspired me to seek female mentees in my next job.” It additionally fostered optimism about careers in academic medicine and made “me more confident as a female provider.” Community-building inside a protected space ultimately resulted in an overarching sense of *solidarity* among participants who felt the events provided an “opportunity to build each other up,” ultimately helping to “develop plans and approaches moving forward.” This solidarity influenced participant well-being by “validating concerns” and addressing them together.

### Weaknesses and Threats

Responses to “weakness” and “threats” overlapped and resulted in three themes: two internal factors, *participation* and *structure of the events*, and one external factor, *optics*. Scheduling issues and participant engagement were noted as barriers to *participation*. Limited time off and difficulty of “devoting free time to these events,” which were “another evening away” from family and friends in a career that already requires working nights and weekends. Participants observed that if not well *structured*, the events could become “redundant” or lapse into negativity and “complaining.” There was concern about enforcing balanced time for social networking and professional development activities so one did not circumscribe the other. Finally, the *optics* of these events were felt to be a threat with multiple respondents reporting the events were perceived as “girl talk” or “lady’s night” and framed as “special treatment,” running the risk of further ostracizing women. While having a *dedicated space* was noted as a strength, others noted that one “can’t change the culture or system without including the majority.”

### Opportunity

*Expansion* was the primary “opportunity” theme. Some respondents desired greater inclusivity, such as promoting “inter-group dialogue” by including nurses and male physicians at occasional sessions. Increased breadth of topic areas (i.e., financial, research), professional development activities and mentorship were suggested. Expansions into other venues, time frames and scheduling modalities, or ability to bring children to events were recommended as growth opportunities.

## DISCUSSION

This evaluation of an applied educational and mentoring model for female EPs found the program was an overall positive experience for both individuals and the local female EP community. The themes above illustrate not only an enhanced feeling of wellness and solidarity as a result of program participation, but also a change in self-perception, confidence, and optimism that have potential to foment long-term change for female physicians. By including alumnae and faculty as core participants along with residents, opportunities for longitudinal professional development and dynamic mentorship pathways emerge.^32^,^33^

Despite these successes, tension exists between the strengths, weaknesses, threats, and opportunities. The greatest program strengths were driven by a “safe space” format and solidarity building, yet these strengths are simultaneously viewed as possible weaknesses or threats given the siloed format and the optics of “exclusivity” created by this protected environment. Additionally, the main opportunity noted by respondents was a focus on expansion that runs the risk of inadvertently degrading cited strengths, thus emphasizing the care with which this feedback must be implemented.

Although data were collected in our isolated departmental context, we suspect that these challenges are not unique and similar programs could learn from our SWOT analysis to build stronger mentorship programs in their own organizations. To that end, we have included the full three-year curriculum to date (Appendix). Our program format and curricular content comprise an easily applied model, which uses existing intellectual and social capital to serve female physicians at all levels.

## LIMITATIONS

This single-center study was conducted over one year with a small sample size of mixed career level EPs. The survey format, rather than interview, may have resulted in limited, superficial responses. Additionally, by surveying invited participants, not just those who attended regularly, we lose some depth of analysis of the participant experience but gain insight into the threats and weaknesses faced by the program. Implicit bias from personal values of the study team may have influenced the thematic analysis. To combat this potential bias, we used consensual coding including male study-team members. Finally, “outcome” data related to physician wellness and other experiences of gender in the workplace were not evaluated.

## CONCLUSION

This evaluation of the Women’s Night curriculum demonstrates it was a positive addition to the department, providing longitudinal opportunities for professional development. Sharing not only the program strengths, but also identified weaknesses, threats, and opportunities for improvement allows others to learn from this experience and implement similar models.

## Supplementary Information



## Figures and Tables

**Table:** Themes identified in participant responses with representative quotes.

Domain	Theme	Sample quotes
Strengths		
	Dedicated space	“Safe space to discuss the realities of being a female physician and have an avenue to ask questions/gain mentorship from female physicians who have been in our shoes.” “Open atmosphere; able to talk about tough topics specific to women in EM in a non-judgmental atmosphere and learn from other women’s experiences.”
	Networking community	“Form stronger relationships with excellent female role models.” “The emphasis on professional development and happiness based on understanding the differences and challenges faced by women.” “Networking with like-minded professional women”
	Solidarity	“In a general sense, it has definitely improved my wellness, and I definitely feel it has improved solidarity and attitudes among the women physicians at our institution, promoting a culture of supportiveness.” “Listening to other women bearing their vulnerabilities is interesting and lends itself for the opportunity to help buttress our sisters’ confidence and understanding of our positions in the EM community.” “Makes the residency feel smaller and creates a tight knit community that has similar experiences and setbacks.”
	Forward guidance	“Moving forward I think women’s night has inspired me to seek female mentees in my next job. It’s so important to have mentors and these nights made me realize that I wish I had sought those relationships earlier in residency.” “A lot of concerns about my future were validated by faculty members and it was reassuring to have a way to discuss these concerns and ways to approach different issues.” “I’m more hopeful about my future in academics. It sounds crazy, but knowing that highly accomplished attendings still feel inadequate made me feel a lot better about myself and gave me a lot more confidence.”
Weaknesses and threats		
	Participation	“We work nights and PM shifts which take us away from family and friends. This is another evening away.” “Scheduling is difficult--both having shifts scheduled and devoting free time to these events.”
	Structure of events	“When the events are less structured, we have a tendency to lapse into just spending the time complaining.” “If I had to choose a weakness, I would say trying to balance the time for social interaction and formal professional discussion; ideally would be 50-50.”
	Optics	“Perception by non-participants that it is exclusionary or lack of recognition of importance of women’s night (ie, lack of recognition of gender gap and the benefit of focused mentoring).” “Perceived as ‘lady’s night,’ or ‘girl talk’ by others.” “A few men expressed that these events were set up because women can’t deal with the pressures of being a ‘real doctor’. While I understand that these individuals have this opinion whether or not we have women’s night, the fact that we had these nights led to this viewpoint being openly discussed in the workplace. It seems like almost every time we had a women’s night, a comment like this would come up.”
Opportunities		
	Expansion	“Continue expanding conversation with different topics and evolving the nights into long-term mentorships that creates projects that residents can collaborate with attendings on during residency.” “One thing that is often brought up is the relationship of the female nurses in the ED…to female residents/attendings vs male. I think this relationship could be greatly improved if 1–2 times per year, we had an event that included the rest of the female staff…not just the physicians.” “Consider inter-group dialogue/inclusion with select male residents.”
