# Supplementary material for: Women’s Night in Emergency Medicine Mentorship Program: A SWOT Analysis
Source: West J Emerg Med. 2019 Dec 18;21(1):37–41. doi: 10.5811/westjem.2019.11.44433 (PMC6948687; doi:10.5811/westjem.2019.11.44433)
Supplement: Supplementary file 1 [file wjem-21-37-s001.docx]

**Appendix A: Women’s Night 3-Year Curriculum**

| **Session** | **Activity** | **Host** |
| --- | --- | --- |
| July 2016 | Welcome Event & Journal Club  Journal Club Topic: The Confidence Gap | Local Restaurant |
| August 2016 | City Wide Women in EM, Outside Guest Speaker: Leader in Emergency Medicine | Local Restaurant |
| September 2016 | Journal Club Topic: Emotional Labor | Faculty |
| January 2017 | Creating Your Elevator Pitch, Outside Guest Speaker: Director of Marketing | Faculty |
| April 2017 | Integrating Medicine and Family Life | Resident |
| May 2017 | Journal Club Topic: Imposter Syndrome |  |
| June 2017 | Reflections on the Year/Planning Ahead | Faculty |
| December 2017 | Holiday Social Event | Resident |
| February 2018 | Alumnae Networking | Resident |
| April 2018 | Navigating Stereotypes | Local restaurant |
| June 2018 | Finances for Physicians | Alumna |
| July 2018 | City Wide Women in EM, Professional Mixer | Local restaurant |
| September 2018 | Improvisation Workshop: Developing Your Voice  Outside Guest Speaker: Bioethicist | Campus |
| October 2018 | Leaders as Catalysts, Outside Guest Speaker: Northwestern School of Management | Faculty |
| November 2018 | Holiday Social Event | Faculty |
| January 2019 | Career Development Workshop | Faculty |
| February 2019 | Resilience in the Face of Professional Setbacks | Faculty |
